# Re: Subramanian and Kumar. Vaccination rates and COVID-19 cases

**DOI:** 10.1007/s10654-021-00816-7

**Published:** 2021-12-24

**Authors:** Emilio Gianicolo, Daniel Wollschläger, Maria Blettner

**Affiliations:** 1 Working Group for the Evaluation of Political Intervention, Division of Epidemiology and Health Services Research, Epidemiology and Informatics (IMBEI), Institute of Medical Biostatistics, University Medical Center of the Johannes Gutenberg-University Mainz, Obere Zahlbacher Str. 69, 55131 Mainz, Germany; 2 Istitute of Clinical Physiology, National Research Council, Lecce, Italy

We have read with interest the correspondence by Subramanian and Kumar [1] and found some major methodological issues which are worth discussing. The authors use an ecological approach to investigate the association of the percentage of population fully vaccinated with the trend in newly reported cases of positive SARS-Cov-2 tests between two consecutive 7-day time periods.

i. Basing the analysis entirely on data from two weeks instead of using the complete time-period since the beginning of the vaccination appears arbitrary.
ii. The analysis does not assess the size of the change in the number of positive test reports between the two time periods, but only evaluates whether a region had reported an increase in positive tests or not.
iii. Another issue arises in comparing countries with enormous differences in terms of testing capacities and/or strategies, availability of vaccines, socioeconomic factors and demographic structures of the populations. One way to overcome this limitation could have been the stratification of the results according to continent awhile accounting for age. The lack of presentation of results makes their interpretation very challenging.
iv. Citing preliminary data from the CDC [2], the authors report an increase in the rates of hospitalizations and deaths amongst the fully vaccinated. However, this representation is incorrect as the CDC report rather evaluates the proportion of fully vaccinated among those hospitalized. The latter proportion is expected to rise as the number of fully vaccinated people increases. Furthermore, this statistic is subject to Simpson’s paradox as the vaccination rate among the elderly is particularly high, as is their risk for severe COVID-19 disease [3].

We agree that vaccination alone does not suffice as strategy to control the spread of the COVID-19 pandemic which instead requires integrating several measures [4]. However, since health outcomes among vaccinated and unvaccinated are not compared in a controlled individual-level study, increasing rates of deaths/hospitalizations even though vaccination rates improve does not, by itself, demonstrate a reduced efficacy of vaccines. In ecological studies, confounders and within-group misclassification may dilute, inflate, or even reverse any association. None of these limitations are mentioned or discussed.

In conclusion, although the authors correctly point out that vaccination alone may not be sufficient to reduce infection rates, their conclusions are not justified by their analysis. Further epidemiological evidence with individual information is needed to examine the efficacy of COVID-19 vaccination in real world studies.
